# Pathogenomic analyses of *Mycobacterium microti,* an ESX-1-deleted member of the *Mycobacterium tuberculosis* complex causing disease in various hosts

**DOI:** 10.1099/mgen.0.000505

**Published:** 2021-02-02

**Authors:** Mickael Orgeur, Wafa Frigui, Alexandre Pawlik, Simon Clark, Ann Williams, Louis S. Ates, Laurence Ma, Christiane Bouchier, Julian Parkhill, Priscille Brodin, Roland Brosch

**Affiliations:** ^1^​ Institut Pasteur, Unit for Integrated Mycobacterial Pathogenomics, CNRS UMR 3525, Paris 75015, France; ^2^​ Public Health England, Porton Down, Salisbury SP4 0JG, UK; ^3^​ Amsterdam UMC, University of Amsterdam, Department of Experimental Immunology, Amsterdam institute for Infection & Immunity, Meibergdreef 9, Amsterdam, Netherlands; ^4^​ Institut Pasteur, Biomics, C2RT, Paris 75015, France; ^5^​ Wellcome Sanger Institute, Wellcome Genome Campus, Hinxton CB10 1SA, UK; ^6^​ Department of Veterinary Medicine, University of Cambridge, Cambridge CB3 0ES, UK; ^7^​ CIIL - Center for Infection and Immunity of Lille, Université de Lille/CNRS UMR 9017/INSERM U1019/CHU Lille/Institut Pasteur de Lille, Lille 59000, France

**Keywords:** ESX-1, mouse and guinea pig models, *Mycobacterium microti*, protective efficacy, virulence evaluation, whole-genome sequencing

## Abstract

*
Mycobacterium microti* is an animal-adapted member of the *Mycobacterium tuberculosis* complex (MTBC), which was originally isolated from voles, but has more recently also been isolated from other selected mammalian hosts, including occasionally from humans. Here, we have generated and analysed the complete genome sequences of five representative vole and clinical *M. microti* isolates using PacBio- and Illumina-based technologies, and have tested their virulence and vaccine potential in SCID (severe combined immune deficient) mouse and/or guinea pig infection models. We show that the clinical isolates studied here cluster separately in the phylogenetic tree from vole isolates and other clades from publicly available *M. microti* genome sequences. These data also confirm that the vole and clinical *M. microti* isolates were all lacking the specific RD1^mic^ region, which in other tubercle bacilli encodes the ESX-1 type VII secretion system. Biochemical analysis further revealed marked phenotypic differences between isolates in type VII-mediated secretion of selected PE and PPE proteins, which in part were attributed to specific genetic polymorphisms. Infection experiments in the highly susceptible SCID mouse model showed that the clinical isolates were significantly more virulent than the tested vole isolates, but still much less virulent than the *M. tuberculosis* H37Rv control strain. The strong attenuation of the ATCC 35872 vole isolate in immunocompromised mice, even compared to the attenuated BCG (bacillus Calmette–Guérin) vaccine, and its historic use in human vaccine trials encouraged us to test this strain’s vaccine potential in a guinea pig model, where it demonstrated similar protective efficacy as a BCG control, making it a strong candidate for vaccination of immunocompromised individuals in whom BCG vaccination is contra-indicated. Overall, we provide new insights into the genomic and phenotypic variabilities and particularities of members of an understudied clade of the MTBC, which all share a recent common ancestor that is characterized by the deletion of the RD1^mic^ region.

## Data Summary

Illumina sequencing data have been deposited in the European Nucleotide Archive (ENA) database under the study PRJEB2091 for *Mycobacterium microti* strains OV254 (ERR027294), ATCC 35782 (ERR027295), 94-2272 (ERR027298) and Maus IV (ERR027297); and under the study PRJEB40482 for *M. microti* strain Maus III (ERR4618952).

Whole-genome sequences of *M. microti* strains have been deposited in the ENA database under the study PRJEB40500 and the following assembly accession numbers: OV254, GCA_904810325; ATCC 35782, GCA_904810335; 94-2272, GCA_904810365; Maus III, GCA_904810345; Maus IV, GCA_904810355.

**Impact Statement**

*
Mycobacterium microti* is a natural ESX-1-deleted member of the *Mycobacterium tuberculosis* complex, which was originally identified as a disease-causing agent in voles, but has since then also been isolated from other mammalian hosts including, in rare cases, humans. To gain new insights in this particular understudied clade, we have characterized here five representative *M. microti* strains of vole and human origin by using whole-genome comparisons, protein secretion analyses and virulence assays in immunocompromised mice. Consistent with the lack of a functional ESX-1 type VII secretion system, we observed that the human *M. microti* isolates were less virulent than *M. tuberculosis* in mice, but more virulent than the attenuated *Mycobacterium bovis* BCG (bacillus Calmette–Guérin) strain, while the vole *M. microti* isolates were even less virulent than BCG in this model. Given that selected *M. microti* strains have been used in the past as successful anti-tuberculosis vaccines, we further investigated the vaccination potential of one such *M. microti* vole isolate in a guinea pig model and found that it induced similar protection as BCG. Combined with the additional attenuation over BCG, this strain might represent a possible alternative anti-tuberculosis vaccine for immunocompromised individuals, for whom BCG vaccination is not recommended.

## Introduction

Within the *Mycobacterium tuberculosis* complex (MTBC)*, Mycobacterium microti* is one of the earliest described members and belongs to the animal-adapted lineage [1, 2]. As indicated by its name, this mycobacterium was first isolated in the 1930s by Dr Wells from diseased voles of the species *Microtus agrestis*, which showed tuberculosis-like lesions [3]. Since then, this subtype of the tubercle bacillus has been identified in field and bank voles, wood mice and shrews, suggesting that wild rodents represent the main reservoir of *M. microti* [3–5]. However, *M. microti* was also found to sporadically infect various other domesticated and wild mammals, including cats [6, 7], dogs [8], pigs [6], wild boars [9], llamas [10, 11], squirrel monkeys [12] and meerkats [13]. Nevertheless, many of these additional host species might represent spillover infections potentially linked to rodents, especially for cats [7], which might occur from environmental contamination via sputum and saliva, or direct contact through skin lesion, bite or feeding [14].

In the late 1990s and early 2000s, *M. microti* infections have also been reported as the cause of human tuberculosis (TB) cases, as *M. microti* was the only mycobacterium that was isolated from the lungs of human immunodeficiency virus (HIV)-positive [15, 16] and HIV-negative [17, 18] TB patients. However, human TB cases caused by *M. microti* infections remain sparse and no evidence of direct human-to-human transmission is available, although it has been suggested [17]. To our knowledge, only a few dozen human TB cases caused by *M. microti* have been reported so far, mainly in European countries, such as The Netherlands [16, 17, 19, 20], the UK [21–24], Germany [15, 18, 25, 26], Switzerland (discussed by Cavanagh *et. al* [5]), France [27] and the Czech Republic [28], but also recently in South Africa, where among the spoligotypes of 215 tested human isolates, 4 corresponded to *M. microti *-specific patterns [29]. Yet, prevalence of *M. microti* infections among humans might be underestimated due to the extremely slow growth of this mycobacterium in diagnostic culture media (6–8 weeks vs 2–4 weeks for *M. tuberculosis *), and due to the difficulty of distinguishing it from *M. tuberculosis* if no genotyping is performed during TB diagnosis in addition to traditional bacteriological tests [17].

Interestingly, *M. microti* lacks a functional ESX-1 type VII secretion system due to a conserved deletion of the *M. microti *-specific region of difference 1 (RD1^mic^) [7, 30–32], which partially overlaps with the RD1^BCG^ region that is absent from the *Mycobacterium bovis* BCG (bacillus Calmette–Guérin) strain [33, 34]. In the past, some of the *M. microti* strains were actually used as live attenuated vaccines against TB in humans in the UK and the former Czechoslovakia [35–37]. In these large-scale vaccination trials, involving more than 5000 adolescents in the UK and more than 500 000 new-borns in the city of Prague between 1950 and 1969 [37], *M. microti *-immunized individuals were protected to a similar extent as individuals vaccinated with BCG [36, 37]. While the *M. microti* strain used in the UK was described as ‘the vole bacillus’ [36], the *M. microti* strain used in Prague was known as the ‘M.P.’ strain, referring to the abbreviated form of ‘Murinus Praha’. The M.P. strain originates from Dr Wells’ OV166 strain, isolated from Orkney voles [3], which was then adapted by Dr Sula to fully synthetic medium and used for preparation of the different vaccine lots for human vaccination [37, 38]. This strain was later also deposited in the American Type Culture Collection (ATCC) and was previously available under the reference number ATCC 35872 (Fig. S1, available with the online version of this article).

In the current study, we sought to investigate the genomic diversity and the virulence spectrum inside the clade of *M. microti* strains. To this end, we selected two *M. microti* vole isolates, namely the widely used laboratory strain OV254 [39–43] and the previously used vaccine strain ATCC 35872 [37], as well as three clinical *M. microti* isolates, and combined whole-genome sequencing approaches, biochemical analysis of protein secretion, virulence evaluation in murine models and preclinical vaccination experiments. We first generated the complete, whole-genome sequences of the five selected *M. microti* strains by using PacBio- and Illumina-based technologies. We then tested selected *M. microti* strains for their virulence potential in severe combined immune deficient (SCID) mice. Finally, we used the ATCC 35782 M.P. vaccine strain for vaccination experiments in a guinea pig model, which also included animals vaccinated with an ESX-1-complemented version of ATCC 35782, named ATCC 35872::ESX-1, as well as BCG-Danish-vaccinated and unvaccinated control animals. Taken together, we explore several key aspects of the clade of *M. microti* to provide new insights into genomic organization, virulence and protective potential of this highly interesting clade of the MTBC.

## Methods

### Bacterial strains

*M. microti* OV254, which was originally isolated from voles in the UK in the 1930s [3], was obtained from M. J. Colston, MRC-NIMR, Mill Hill, London, UK. *M. microti* 94-2272 was isolated in 1988 from the perfusion fluid of a 41-year-old dialysis patient [17] and was kindly provided by L. M. Parsons, Wadsworth Center Albany, Albany, NY, USA. *M. microti* ATCC 35782 was previously purchased from the ATCC [designation: TMC 1608 (M.P. Prague)] (Fig. S1). *M. microti* Maus III (also known as strain 6740/00 or B3) and *M. microti* Maus IV (also known as strain 1479/99 or B4) were obtained from the collection of the National Reference Center for Mycobacteria, Forschungszentrum Borstel, Germany [30]. Typically, *M. microti* strains were grown either on Middlebrook 7H9 liquid medium (Difco) containing 10 % (v/v) acid-dextrose-catalase (ADC; Difco), 0.2 % (w/v) pyruvic acid and 0.05 % (v/v) Tween 80, or on Middlebrook 7H11 solid medium (Difco) supplemented with 10 % (v/v) oleic acid-dextrose-catalase (OADC; Difco).

### Whole-genome sequencing data generation

Genomic DNA of *M. microti* strains was isolated by bead-beating, boiling and ethanol precipitation, as described previously [44]. Genomic DNA was processed using the SMRTbell template library preparation kit and sequenced at the Biomics platform of the Institut Pasteur (Paris, France) using the PacBio SMRT long-read sequencing technology (Chemistry v2.1; Sequel ICS v5.0.1; SMRT Link v5.0.1). In parallel, genomic DNA was also sequenced using the Illumina paired-end short-read sequencing technology. Strains OV254, ATCC 35782, 94-2272 and Maus IV were sequenced at the Wellcome Trust Sanger Institute (Hinxton, UK) using a Genome Analyzer IIx device, while strain Maus III was sequenced at the Biomics platform of the Institut Pasteur (Paris, France) using a HiSeq 2500 device. Sequencing read datasets of additional *M. microti* strains for phylogenomic analyses were downloaded from the Sequence Read Archive (SRA) database: strain ATCC 19422 (also known as strain ‘12’; run SRR3647357) [45]; strains isolated from wild boars in Italy G25821 to G25828 (runs from ERR2659163 to ERR2659171) [46]; and llama-type strains 416/01 and 8753/00 (runs ERR553376, ERR553377 and from ERR551111 to ERR551113) [47].

### Whole-genome assembly

Raw PacBio subreads were assembled using Canu v1.7 [48] (parameters: genomeSize=4.4m; -pacbio-raw). Generated supercontigs were then organized as compared to the genome of *M. bovis* BCG Pasteur 1173P2 (AM408590) [49] using MeDuSa v1.6 [50] (default parameters). Illumina read pairs were first trimmed using Trimmomatic v0.36 [51] (parameters: LEADING 20; TRAILING 20; SLIDINGWINDOW windowSize 5, requiredQuality 20; MINLEN 50; AVGQUAL 20). Complete read pairs were then assembled using SPAdes v3.10.1 [52] (parameters: --careful; -k 21,33,55; --phred-offset 33) and providing either raw PacBio subreads (parameter: --pacbio) or the scaffolds of PacBio supercontigs (parameter: --trusted-contigs). Illumina contigs thus generated were then compared to the PacBio scaffolds using blastn from blast+ v2.5.0+ [53] (parameters: -perc_identity 95; -dust no; -soft_masking no) to correct erroneous arrangements and to fill undefined gaps.

### Gene annotation

Whole-genome assemblies of *M. microti* strains were annotated using Prokka v1.13.4 [54] (parameters: --addgenes; --kingdom Bacteria; --gcode 11; --rfam; --rnammer). Genome annotation was performed against the default bacterial core databases, as well as against a custom Genus database (parameter: --usegenus) composed of annotated genes from *M. tuberculosis* H37Rv (AL123456), *M. bovis* AF2122/97 (LT708304) and *M. bovis* BCG Pasteur 1173P2 (LT708304) [49, 55, 56]. Prediction and synteny analysis of orthologous genes was performed according to the Synima pipeline r20122018 [57]. Briefly, nucleotide sequences of all annotated protein-encoding genes were first compared using blastn from blast+ v2.2.26 [53]. Groups of orthologous genes were then determined from nucleotide alignments using OrthoMCL v1.4 [58], and chains of syntenic genes were retrieved using DAGchainer r02062008 [59]. Hierarchical clustering of orthologous genes was performed using the Ward’s D2 minimum variance method on asymmetric binary (Jaccard) distances.

### Variant calling

Illumina read pairs were mapped against a given reference genome using bwa-mem v0.7.15 [60] (default parameters). Mapped reads were first locally realigned around indels using Picard Tools v2.8.1 (http://broadinstitute.github.io/picard/) and GATK RealignerTargetCreator and IndelRealigner tools v.3.7.0 [61] (default parameters). Read alignments with a mapping quality lower than one and flagged as secondary were then discarded using SAMtools view v1.3.1 [62] (parameters: -q 1; -F 256). Duplicated read pairs were also removed from the alignment map using an in-house Python script to prevent the presence of potential amplification contaminants. Two read pairs were considered as a duplicate if they mapped at the same positions, had an identical sequence and stemmed from the same DNA strand. In the case of duplicates, the pair with the highest base-quality mean was kept. The resulting alignment map was finally converted into pileup format using SAMtools mpileup v1.3.1 [62] (parameters: -B; -Q 0; -s). SNPs and indels were called using VarScan pileup2snp and pileup2indel v2.4.2 [63], respectively. To detect and correct sequencing errors within PacBio whole-genome assemblies, variants were called using a minimum read depth of 5 and a minimum variant frequency of 51 %. To identify genetic variations between two different strains, the called variants were filtered according to the VarScan detection criteria with the following adjustments to increase stringency: (i) a minimum read depth of 5; (ii) a minimum variant frequency of 80 %; (iii) a minimum mean base quality of 20; and (iv) a minimum variant support on each strand of 25 %. Effects of called SNPs and indels on annotated genes were predicted using SnpEff ann v4.3i [64] (parameters: -no-upstream; -no-downstream). Variant intersections were determined using the R package UpSetR v1.3.3 [65]. Functional annotation and categorization of genes affected by variants was performed using RAST [66]. Density of mapped reads and filtered variants was calculated in 5-kb non-overlapping windows and plotted using Circos v0.69-6 [67].

### Phylogenetic reconstruction

All variants detected for each *M. microti* strain against *M. tuberculosis* H37Rv were first filtered according to the reference annotation (AL123456.3) to remove those located within repetitive sequences, namely genes encoding proteins of the Pro-Glu (PE), Pro-Pro-Glu (PPE) and PE_PGRS (Polymorphic GC-rich Sequence) families, mobile elements and repeat regions. Sequencing read depth at positions of the remaining variants was then parsed among all alignment maps to exclude variants for which the read coverage was lower than the minimum threshold fixed for variant calling (<5 mapped reads) in at least one strain. Remaining variants were finally used to produce a phylip alignment that contained only variable sites (SNPs). This resulting alignment, excluding ambiguous variants and indels, accounted for 74.7 % of the initial polymorphic sites. Maximum-likelihood phylogenetic reconstruction was inferred using RAxML v8.2.12 [68] with the GTR substitution model, GAMMA distribution of rate heterogeneity, Lewis’ correction of ascertainment bias and 1000 rapid bootstrap replicates (parameters: -m ASC_GAMMA; --asc-corr=lewis; -f a). Bipartition support of the best-scoring tree rooted using *M. tuberculosis* H37Rv was computed according to the transfer bootstrap expectation (TBE) from booster v0.1.2 [69]. The resulting maximum-likelihood phylogenetic tree that is shown was drawn and annotated using the R package ggtree v1.14.6 [70].

### Secretion analysis and immunoblots

Secretion analysis was performed as described elsewhere [71, 72]. Briefly, strains ATCC 35782, 94-2272, Maus III, OV254 and OV254-C (OV254 transduced with the pMV::mt2419-22 vector that encodes the *ppe38-71* genetic locus [73, 74]) were pre-cultured in general culture medium until an OD_600_ of approximately 1.0. At this point, cultures were washed three times in secretion medium, consisting of 7H9 medium without ADC, supplemented with 0.2 % (w/v) dextrose, 0.2 % (w/v) pyruvic acid and 0.025 % (v/v) Tween 80. Washed cells were inoculated at an OD_600_ of 0.5 in secretion medium and were incubated 48 h at 37 °C. These cultures were harvested by centrifugation and the supernatant was filtered using a 0.22-µm filter (Millipore) and precipitated with trichloroacetic acid, before being resuspended and boiled 10 min at 95 °C in solubilization/denaturation buffer. Cells were washed with PBS (1x) and resuspended in solubilization/denaturation buffer before being heat-inactivated for 3 h at 80 °C, followed by sonication to disrupt cells and boiling for 10 min at 95 °C. Protein samples were loaded on SDS-PAGE gels with an equivalent of 0.225 OD units for whole cell lysates and 0.6 OD units for culture filtrates. Western blots were stained with anti-PGRS antibody 7C4.1F7 (a kind gift of the antibody-producing clone was from M.J. Brennan, Aeras, Rockville, MD, USA; and of purified antibody from W. Bitter, Amsterdam UMC, Amsterdam, The Netherlands) [75]; anti-SigA (a kind gift from I. Rosenkrands, Statens Serum Institut, Copenhagen, Denmark); rabbit polyclonal anti-EsxN (rMtb9.9A) [76]; and rabbit polyclonal anti-PPE41 (a kind gift from W. Bitter, Amsterdam UMC, Amsterdam, The Netherlands) [77].

### Virulence assay in SCID mice

For sample preparation, 50 ml cultures of the individual mycobacterial strains were grown in parallel in Middlebrook 7H9 medium supplemented with 10 % (v/v) ADC, 2 % (w/v) pyruvic acid and 0.05 % (v/v) Tween 80. Bacteria were harvested, washed and resuspended in 50 mM sodium phosphate buffer (pH 7.0). Bacterial suspensions, obtained by brief sonication with disinfected, sterile equipment were then aliquoted and frozen at −80 °C. A single defrosted aliquot was used to quantify the c.f.u. of stock solutions. A typical concentration of stock solutions was 10^9^ c.f.u. ml^−1^. For virulence studies, 13 6-week-old female SCID mice (Charles River) per group were infected intravenously via the lateral tail vein with 1×10^6^ c.f.u. in 200 µl of PBS (1x). After 1 day of infection, the delivered dose was determined from spleens from three representative control mice per group. Organs were homogenized using an MM300 (Qiagen) apparatus and 2.5-mm diameter glass beads. Serial fivefold dilutions in PBS (1×) were plated on Middlebrook 7H11 OADC agar and c.f.u. were counted after 3–6 weeks of growth at 37 °C, depending on the strain. The weight of the 10 remaining mice per group was monitored over several weeks and mice were killed when reaching the humane endpoint, defined as a loss of >20 % of bodyweight in accordance with ethics committee guidelines. Sample size choice was determined by taking into account the rule of 3Rs (replacement, reduction, refinement) and statistical requirements. Statistical significance in survival between groups of infected animals was assessed with the log-rank (Mantel–Haenszel) test using the R package survival v3.2-7 [78]. For the pilot virulence assay, five SCID mice (Charles River) per group were infected intravenously and the delivered dose was determined from the spleen from one mouse per group on the day of infection.

### Assessment of protective efficacy induced in guinea pigs

A low-dose aerosol guinea pig infection model was used as previously described [79, 80]. Dunkin Hartley guinea pigs free from pathogen-specific infection (weight 250–350 g) were randomly assigned to vaccine groups and identified using subcutaneously implanted microchips (Plexx) to enable blinding of the analyses wherever possible. Statistical power calculations (Minitab, version 16) were performed to determine group sizes using previous data (SD, approximately 0.5) to reliably detect a difference of 1.0 log_10_ in the group median number of c.f.u. ml^−1^. Guinea pigs (eight per group) were vaccinated once subcutaneously on the nape with 5×10^4^ c.f.u. in 250 µl of PBS (1x) *M. microti* strain ATCC 35782, ATCC 35782::ESX-1 or BCG Danish, or inoculated with saline as a control. At 12 weeks after immunization, animals were challenged by the aerosol route with *M. tuberculosis* H37Rv NCTC 7416 (retained dose 10–20 c.f.u./lungs). Protection was determined by measuring the bacterial load 4 weeks after challenge, when guinea pigs were killed by an overdose with sodium pentobarbital given by the intraperitoneal route. At necropsy, lungs and spleens were removed as described previously [79], and lobes/sections divided between bacteriology and histology analyses. For bacterial load analysis, each tissue was homogenized in 2 ml sterile PBS (1x). Each tissue homogenate volume was recorded and serially diluted in sterile PBS (1x) and 100 µl of each dilution was plated in duplicate onto Middlebrook 7H11 OADC selective agar. Plates were incubated at 37 °C for up to 4 weeks. Following incubation, colonies were enumerated (as c.f.u.), and the concentration of total bacilli in each sample homogenate was calculated. Bacterial load data were expressed as log_10_(total c.f.u.) and statistical significance was assessed with the one-way ANOVA test followed by Tukey HSD post-hoc test using the R package rstatix v0.6.0. For histological analysis, tissue representative of each lung and spleen, sampled consistently between animals, was processed routinely (by formaldehyde fixation) and embedded in paraffin wax. Sections (thickness of approximately 5 µm) were stained with haematoxylin and eosin. The nature and severity of the lesions were assessed by a 'blinded' veterinary pathologist, using a subjective scoring system as described elsewhere [81]. For the spleen, a score, based on number of lesions, was calculated. For the lung, each lobe was assigned a score as follows: 0, normal; 1, very few or small lesions, 0–10 % consolidation; 2, few or small lesions, 10–20 % consolidation; 3, medium sized lesions, 20–33 % consolidation; 4, moderate sized lesions, 33–50 % consolidation; 5, 50–80 % consolidation, extensive pneumonia, >80 % consolidation, plus a number of foci of necrosis. Scores from each lobe were combined and a mean score from lung lobes and spleen was calculated for each group. Group mean histopathology scores were then compared between groups and with bacterial loads and statistical significance was assessed with the Kruskal–Wallis test followed by Dunn post-hoc test with Benjamini–Hochberg correction using the R package rstatix v0.6.0. All vaccine groups, including saline negative controls, showed similar weight profiles during both the vaccination and challenge phases of the study, and no animals reached their humane endpoint prior to the end of the experiment.

## Results

### Whole-genome sequencing of *M. microti* strains

**Fig. 1.**
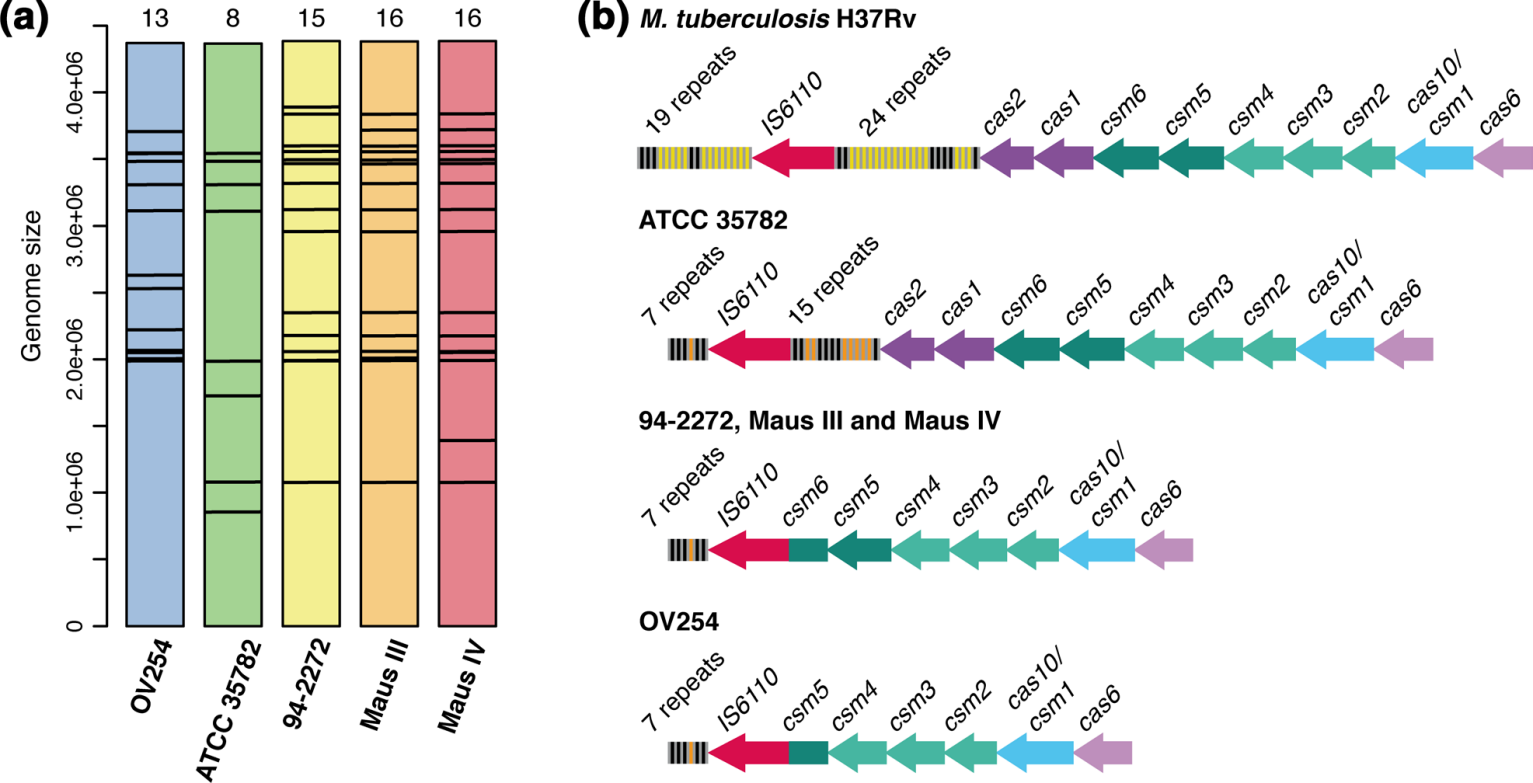
IS*6110* insertion element profiles and type III-A CRISPR-Cas locus in *M. microti* strains. (a) IS*6110* elements (black bars) identified along the genome of each *M. microti* strain. The number of IS*6110* elements is indicated at the top of each coloured bar. The genome size is in bp. (b) Type III-A CRISPR-Cas locus in *M. microti* strains as compared to *M. tuberculosis* H37Rv. Repeats are coloured in grey; spacers that are identical between *M. tuberculosis* H37Rv and *M. microti* strains are coloured in black, while those that are specific either to *M. tuberculosis* H37Rv or to *M. microti* strains are coloured in yellow and orange, respectively.

Using PacBio and Illumina sequencing data, we generated the full-length genome sequences of five *M. microti* strains: OV254 (vole isolate) [42], ATCC 35782 (M.P. vaccine strain) [37], 94-2272 (clinical isolate) [17], Maus III (clinical isolate, also known as strain 6740/00 or B3) [30] and Maus IV (clinical isolate, also known as strain 1479/99 or B4) [30, 82]. Overall, the genomes of the five *M. microti* strains were highly similar in terms of size and global organization (Table 1, Fig. S2), whereby the clinical *M. microti* isolates contained the largest genomes with, on average, an additional 14.1 and 17.5 kb as compared to strains OV254 and ATCC 35782, respectively (Table 1). Some of the most evident differences found corresponded to variations in the insertion sites and the copy number of IS*6110* insertion elements (Fig. 1a, Table 1). Notably, *M. microti* strains depicted differences in their CRISPR-Cas loci that coincided with IS*6110* insertion events (Fig. 1b). ATCC 35782 was the only *M. microti* strain studied here with a potentially intact CRISPR-Cas locus, albeit with shorter repeat/spacer clusters, as compared to the *M. tuberculosis* H37Rv control strain (Fig. 1b). By contrast, the other four *M. microti* strains displayed truncated CRISPR-Cas loci, with both *cas1* and *cas2* genes being missing and *csm6* being either fully or partly deleted (Fig. 1b). A section of the repeat/spacer region was identical between all five *M. microti* strains, for which five out of six spacers matched spacers of *M. tuberculosis* H37Rv, in agreement with previous spoligotyping studies [17, 30, 83]. Further database comparisons showed that the remaining sixth spacer of this region was only detected in a few strains of TbD1-intact *M. tuberculosis* and *Mycobacterium africanum* strains, as well as in *M. microti* ATCC 19422 (also known as strain ‘12’), which is consistent with a previous study that focused on the direct repeat (DR) locus of MTBC members [83]. Strain ATCC 35782 harboured additional spacers and repeats, as compared to the other *M. microti* strains, for which half of them were identical to the ones found in *M. tuberculosis* H37Rv (Fig. 1b), whereas others were mainly detected in TbD1-intact *M. tuberculosis *, *M. africanum *, *M. bovis* and BCG vaccine strains, in agreement with previous spoligotyping studies [17, 30, 83]. Interestingly, the CRISPR-Cas locus of *M. microti* ATCC 35782 appeared identical to the one described for *M. microti* ATCC 19422.

**Table 1.** Genomic characteristics of sequenced *M. microti* strains

Strain	Genome length (bp)	No. of IS*6110* elements	No. of annotated genes	No. of core genes	No. of additional genes	No. of unique genes	No. of called variants
OV254	4 369 915	13	4114	3984	33	16	n/a
ATCC 35782	4 366 549	8	4103	3984	27	11	505
94-2272	4 384 561	15	4130	3994	45	10	484
Maus III	4 382 575	16	4116	3996	37	2	532
Maus IV	4 385 022	16	4137	3992	42	22	516

n/a, not applicable.

### Gene synteny within *M. microti* strains and polymorphisms affecting ESX-5 type VII protein secretion

**Fig. 2.**
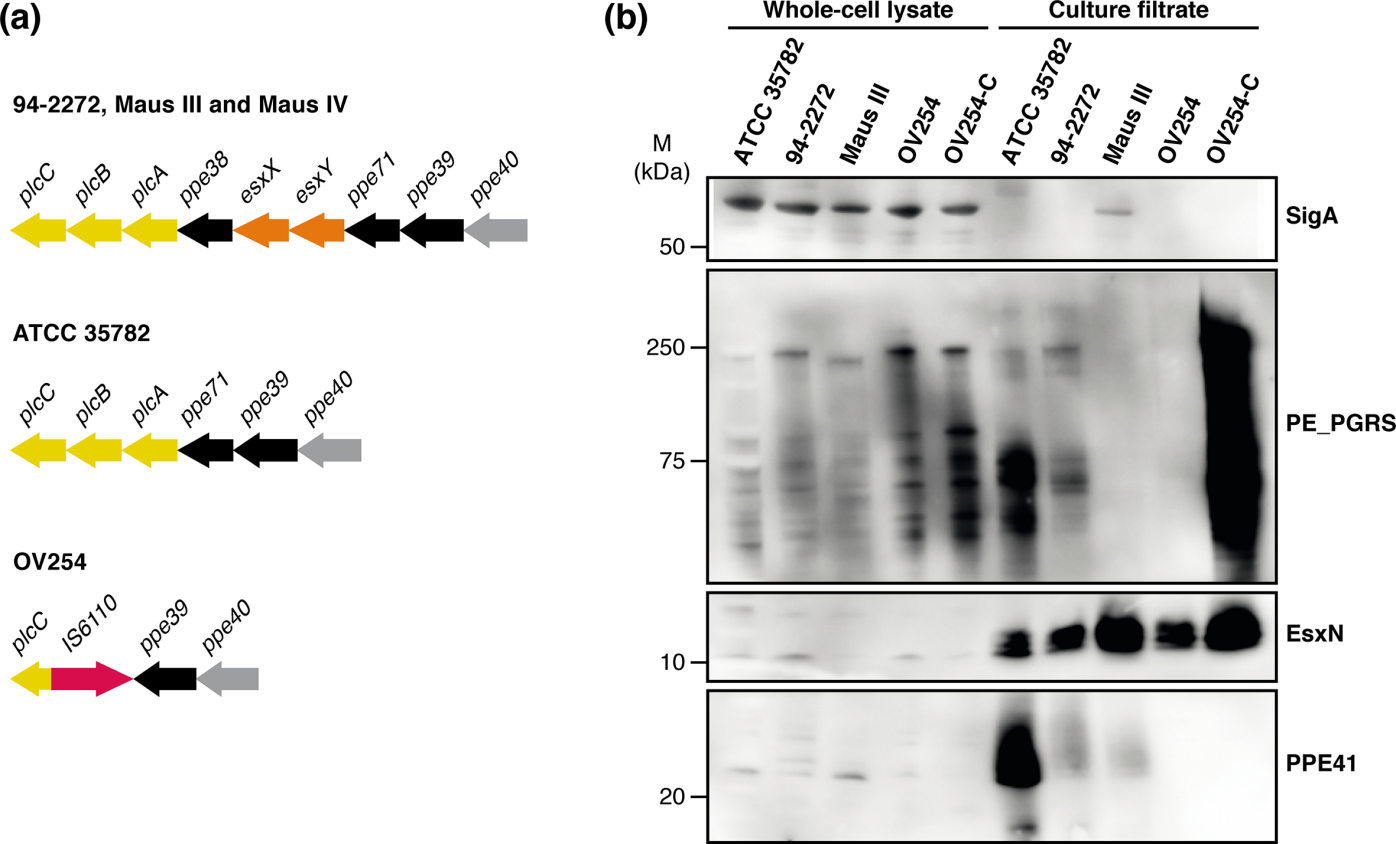
Phospholipase C locus and ESX-5 type VII protein secretion in *M. microti* strains. (a) Phospholipase C locus in *M. microti* strains. (b) Immunoblot secretion analysis of PE_PGRS proteins, PPE41 and EsxN, in *M. microti* strains. OV254-C corresponds to the OV254 strain complemented with the *ppe38-71* locus from *M. tuberculosis *. SigA was used as a loading and lysis control. M, Molecular mass.

To further investigate genomic variability within *M. microti* strains, reconstructed genomes were annotated using bacterial core databases and a customized mycobacterial database (see Methods). On average, 4120 genes were identified for each genome (Table 1), including, on average, 4039 (98.0 %) protein-encoding genes. Following gene annotation, we sought orthologous and non-orthologous genes among the five *M. microti* strains (Fig. S3a, b). Most of the protein-encoding genes (on average 3990, 98.8 %) belonged to a core gene set that was shared by all five *M. microti* strains (Table 1). However, additional orthologues (on average 37; 0.9 %) were only identified in some strains but not in others, and a few genes (on average 12; 0.3 %) were unique to a specific *M. microti* strain (Tables 1 and S1). While most of these unique genes were annotated as encoding hypothetical proteins or PPE/PE_PGRS family proteins, whose functions are still unknown, for some others a more specific role was suggested by the annotation (Fig. S3c, Table S1). As examples, the CRISPR-associated endonuclease genes *cas1* and *cas2* were only found in strain ATCC 35782 (Fig. 1b), while the gene encoding the antitoxin VapB39 was unique to strain 94-2272 (Table S1). Moreover, we found important variations in the locus commonly referred to as RD5 that contains three phospholipase C genes (*plcA-B-C*) as well as the *ppe38-ppe71-esxX-esxY* locus (Fig. 2a), which is in agreement with a previous study that defined this locus as a hypervariable region within the MTBC members [84]. The three clinical strains 94-2272, Maus III and Maus IV harboured a full gene set at this locus that resembled that of most *M. tuberculosis* strains (Fig. 2a). By contrast, the vole strain OV254 showed the smallest locus and lacked *plcA*, *plcB* and part of *plcC*, as well as the *ppe38*, *ppe71*, *esxX* and *esxY* genes (Fig. 2a), a deletion that was previously characterized as RD5^mic^ [30, 84]. As for strain ATCC 35782, the full set of *plcA-B-C* genes was present, as was the single *ppe71* gene, whereas *ppe38, esxX* and *esxY* were absent (Fig. 2a). Importantly, previous reports found that a functional copy of either *ppe38*, or its almost identical homologue *ppe71*, is needed for the export of the so-called PPE-MPTR and PE_PGRS proteins by the cognate ESX-5 type VII secretion system [73, 84]. Deletion of the *ppe38-71* locus made *M. tuberculosis* more virulent in animal models and this deletion occurred at the branching point of the hypervirulent modern Beijing strains [73]. RD5-like deletions, abrogating PE_PGRS/PPE-MPTR secretion, have occurred independently in multiple branches of the animal-adapted MTBC members [72, 84]. In contrast, these deletions are relatively rare in the closely related human-associated *M. africanum* strains of lineages L5 and L6, and have not been described in MTBC members infecting chimpanzees, seals and sea lions [72, 74, 84]. However, we did previously report reduced levels of PE_PGRS secretion and PPE-MPTR immunogenicity in *M. africanum* L5 isolates that did have a functional *ppe38*/*71* copy [72]. Based on these observations, we investigated protein secretion in the *M. microti* strains ATCC 35782, 94-2272, Maus III and OV254. In addition, we introduced the *ppe38-71* locus of *M. tuberculosis* CDC1551 on an integrative plasmid to OV254 (OV254-C), which lacks an endogenous copy of either *ppe38* or *ppe71*. As expected, ATCC 35782 secreted PE_PGRS proteins as well as other ESX-5 substrates, EsxN and PPE41, that are not dependent on *ppe38*/*71* for secretion (Fig. 2b). Inversely, while OV254 did secrete regular levels of EsxN, this strain did not secrete any PE_PGRS proteins, as was expected due to its RD5^mic^ deletion. This phenotype was fully complemented in OV254-C, confirming the *ppe38-71* locus deletion as the cause of this secretion defect. Surprisingly, OV254 also did not secrete the *ppe38*/*71*-independent ESX-5 substrate PPE41 and this phenotype was not affected by complementation of the *ppe38-71* locus. Detailed investigation of the genome sequence of OV254 revealed that this strain has a 2-bp insertion in the coding sequence of *ppe41* (Tables S2 and S3) explaining this phenotype. Finally, both human isolates 94-2272 and Maus III had clearly reduced to fully deficient PE_PGRS secretion and relatively low levels of PPE41 secretion, while the ESX-5 substrate EsxN was unaffected (Fig. 2b).

### Polymorphism signals among *M. microti* strains

**Fig. 3.**
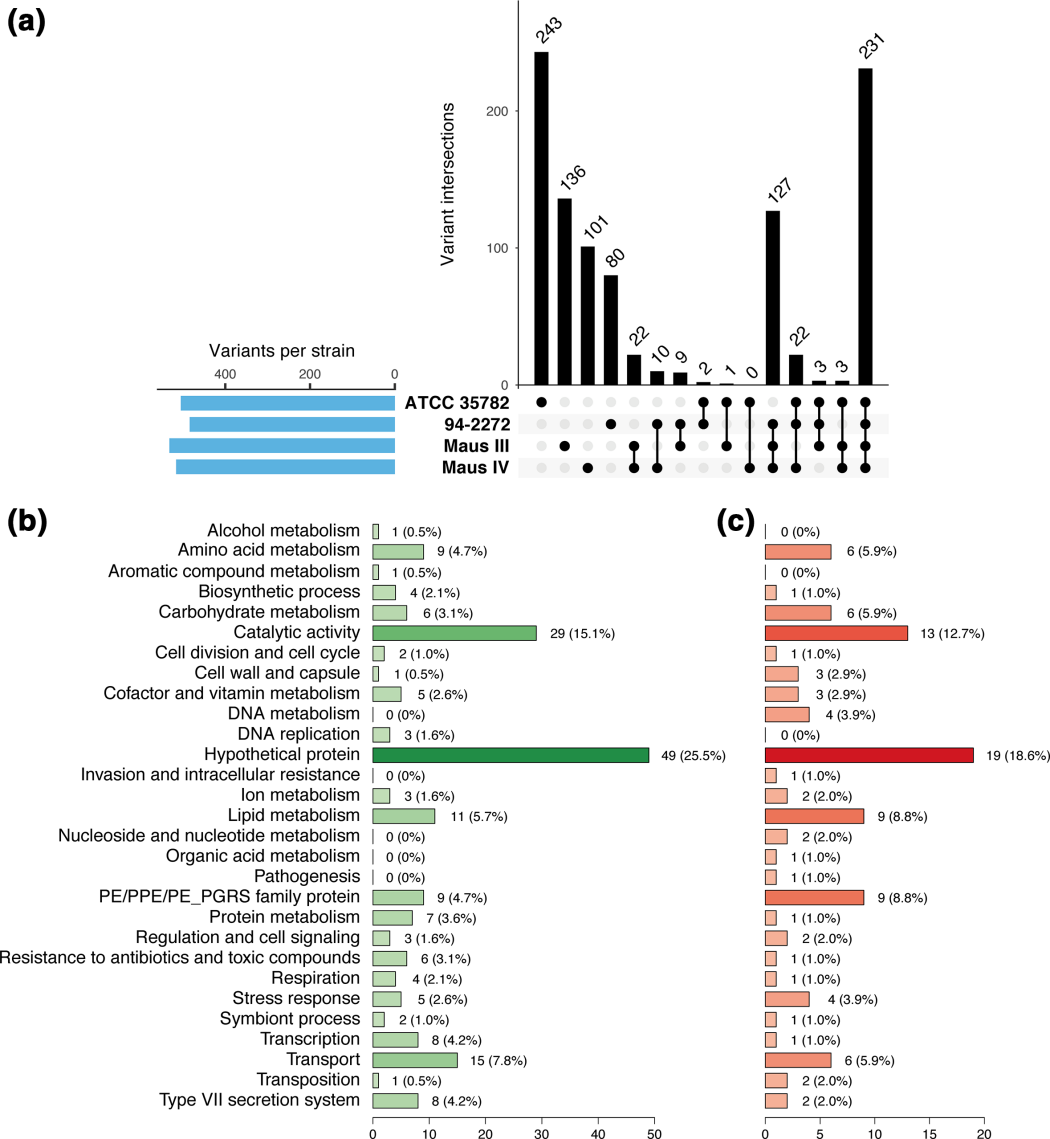
Polymorphism signals detected within *M. microti* strains. (a) Intersections of called variants (blue bars) within *M. microti* strains when compared to the genome of OV254. Black circles in the bottom matrix layout indicate the strains that are part of each intersection. The number of called variants within each intersection set is indicated at the top of each black bar. (b, c) Functional categories of genes affected by variants specific to strain ATCC 35782 (b), and by variants common to strains 94-2272, Maus III and Maus IV (c). The number and percentage of genes within each category are indicated.

Next, we compared the five *M. microti* strains at the base resolution by mapping sequencing reads from each strain against the reconstructed genome of the vole strain OV254, followed by calling variants (SNPs and indels). Between 484 and 532 variants were detected for each *M. microti* strain as compared to OV254, accounting for a total of 990 unique variants (Tables 1 and S2). Among these variants, 231 were shared by the four *M. microti* strains (Fig. 3a), which corresponded to polymorphism signals unique to OV254 as no variants were detected at these positions when mapping OV254 sequencing reads against its own genome. Among the four *M. microti* strains studied, ATCC 35782 depicted the highest number of specific variants (Fig. 3a). The 243 variants specifically detected for this strain affected various genes, notably: eight genes associated with the type VII secretion system, i.e. *espK*, *esxE*, *eccD1* (adjacent to the deleted RD1^mic^ region of difference), *eccC3*, *mycP3*, *eccC5*, *eccD5* and *mycP5*; five genes involved in stress response (such as *fbpB*, *mshA*, *proX* and *sodC*); and six genes participating in resistance to antibiotics and toxic compounds (including *blaC*, *ctpV* and s*tp*) (Fig. 3b, Table S2). Interestingly, the number of specific variants identified for the human isolates 94-2272, Maus III and Maus IV was much more reduced than for strains OV254 and ATCC 35782 (80, 136 and 101, respectively), which was due to the detection of 127 variants that were common to the three clinical isolates (Fig. 3a). Among the genes affected by these common variants, two genes were associated with type VII secretion systems (*eccB5* and *espI*), four genes were involved in stress response (including *pncB2*, *mca* and *deaD*), and one gene was related to processes involved in pathogenesis (*rpfA*) (Fig. 3c, Table S2).

### Genomic variability between *M. microti* strains and *M. tuberculosis*

**Fig. 4.**
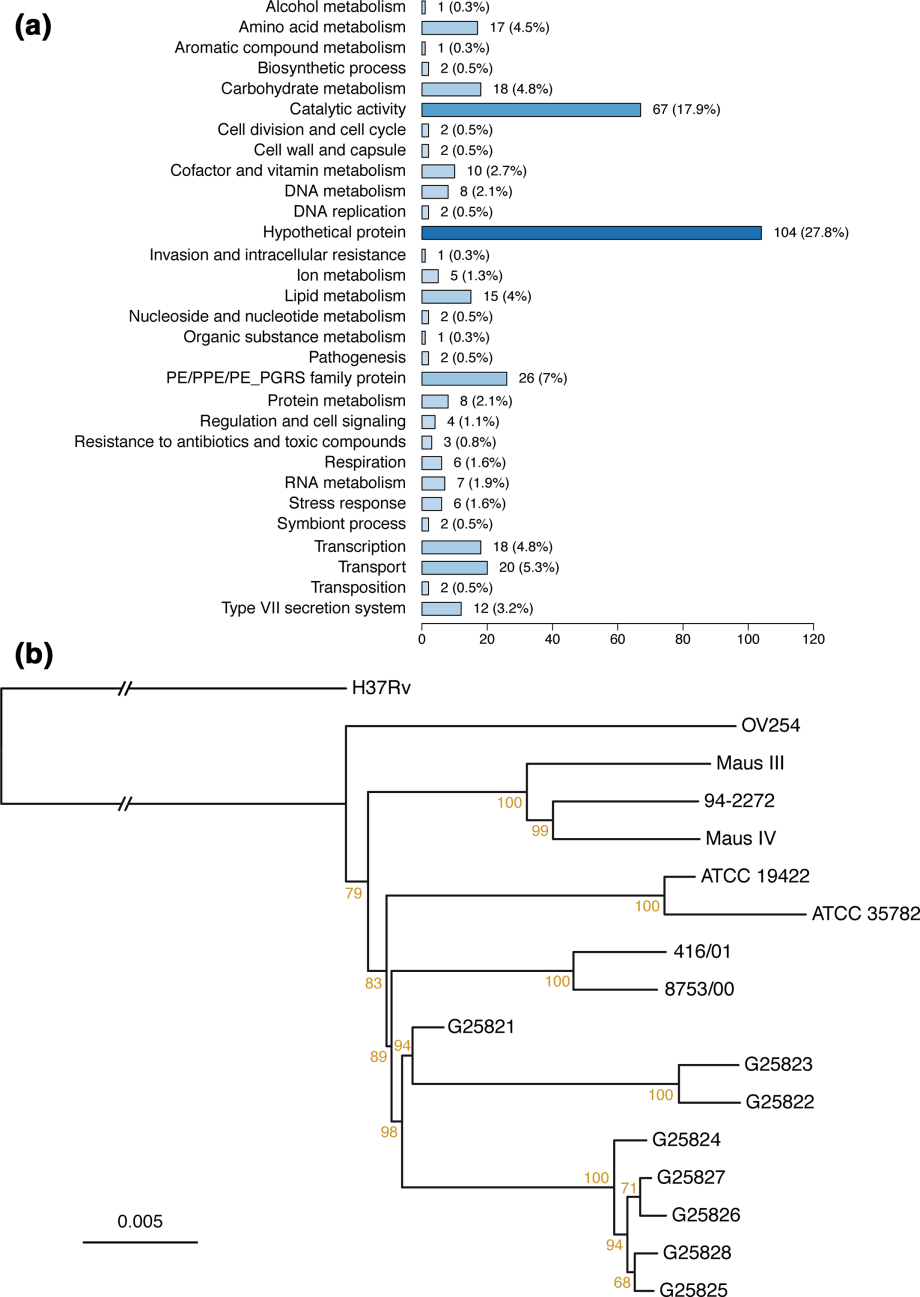
Polymorphism signals detected between *M. microti* strains and *M. tuberculosis* H37Rv. (a) Functional categories of genes affected by variants specific to strain OV254 and/or strain ATCC 35782 as compared to *M. tuberculosis* H37Rv. The number and percentage of genes within each category are indicated. (b) Maximum-likelihood phylogenetic tree of *M. microti* strains. Branch lengths are proportional to nucleotide substitutions detected relative to *M. tuberculosis* H37Rv, used as outgroup strain for phylogenetic reconstruction. Support values obtained from 1000 bootstrap replicates are indicated in brown as percentages. Scale bar represents the number of substitutions per site.

Next, we investigated the genomic variability between *M. microti* and *M. tuberculosis* by aligning sequencing reads from each *M. microti* strain against the reference genome of *M. tuberculosis* H37Rv. The four regions of difference (RDs), from RD7 to RD10, characteristic for *M. africanum* L6 and animal-adapted lineages of tubercle bacilli [46, 85], were deleted in all *M. microti* strains as compared to *M. tuberculosis* H37Rv (Table 2,Fig. S4). In addition, one of both *M. tuberculosis* H37Rv prophage regions, phiRv1 (RD3), was missing in all *M. microti* strains, while the prophage region phiRv2 (RD11) was present (Table 2, Fig. S4) [30]. However, all *M. microti* strains lacked the IS*6110* insertion element present in the RD11 region of *M. tuberculosis* H37Rv. *M. microti* strains were also characterized by missing the RD1^mic^ region (Table 2, Fig. S4), which encompasses a large portion of genes encoding the ESX-1 secretion system, including ESAT-6 family proteins EsxA and EsxB. This genomic region is present in *M. tuberculosis* H37Rv and partly overlaps with the RD1^BCG^ region absent from BCG strains [30, 49]. The MiD3 region was absent from all *M. microti* strains tested, while the presence or absence of regions MiD1 and RD5^mic^, which correspond to type III-A CRISPR-Cas and phospholipase C loci, respectively, was variable among strains (Figs 1b, 2a and S4, Table 2). As for the MiD2 region, it was only absent from strain OV254, but present in all other *M. microti* strains (Table 2, Fig. S4). Moreover, both genes *rv3019c* and *rv3020c*, encoding the ESAT-6 like proteins EsxR and EsxS, respectively, were missing from all five *M. microti* strains, in agreement with previous findings [86]. In addition to these, between 2051 and 2128 variants (SNPs and indels) were detected for each *M. microti* strain relative to *M. tuberculosis* H37Rv. These variants were uniformly detected along the genome of *M. tuberculosis* H37Rv and corresponded to a total of 2962 unique variants (Fig. S5, Table S3). Among these variants, 519 were only detected in OV254 and/or ATCC 35782 strains, but not in human isolates 94-2272, Maus III and Maus IV. These variants were notably enriched in genes associated with type VII secretion systems (*eccA5*, *eccC2*, *eccC3*, *eccD1*, *eccD4*, *eccD5*, *esxE*, *esxL*, *esxN*, *mycP3*, *mycP4* and *mycP5*) and with type II toxin–antitoxin systems (*vapC31* and *vapC37*), as well as in genes involved in DNA metabolism (such as *cas1*, *end* and *uvrC*), in pathogenesis (*hbhA* and *rpfA*), in resistance to antibiotics and toxic compounds (including *blaC* and *ctpV*), in RNA metabolism (such as *rpoB* and *rpoC*) and in stress responses (including *cfp29*, *fbpB*, *mshA*, *proX* and *sodC*) (Fig. 4a, Table S3).

**Table 2.** Regions of difference (RDs and MiDs) in *M. microti* strains

Region	OV254	ATCC 35782	94-2272	Maus III	Maus IV
RD1^mic^	Absent	Absent	Absent	Absent	Absent
RD3	Absent	Absent	Absent	Absent	Absent
RD7	Absent	Absent	Absent	Absent	Absent
RD8	Absent	Absent	Absent	Absent	Absent
RD9	Absent	Absent	Absent	Absent	Absent
RD10	Absent	Absent	Absent	Absent	Absent
MiD3	Absent	Absent	Absent	Absent	Absent
RD11	Partial	Partial	Partial	Partial	Partial
MiD1	Absent	Present	Partial	Partial	Partial
RD5^mic^	Absent	Partial	Present	Present	Present
MiD2	Absent	Present	Present	Present	Present

### Genome-based phylogeny

To assess the genome-based phylogeny of *M. microti* strains relative to *M. tuberculosis *, we included the publicly available whole-genome sequencing data of 11 additional *M. microti* strains of various origins: strain ATCC 19422, which was isolated from a vole by Dr Wells [3]; strains 416/01 and 8753/00 with a llama-type spoligotype; and eight strains named G25821 to G25828 that were isolated from wild boars in Italy between 2003 and 2011 [9]. The results of these comparisons showed that between 2045 and 2323 variants (SNPs and indels) were detected for each of these 11 additional *M. microti* strains as compared to *M. tuberculosis* H37Rv, extending the total number of unique variants to 4019 (Table S3). A maximum-likelihood phylogenetic tree was inferred on all *M. microti* strains from 2993 variable sites covering 3004 unique SNPs and rooted using *M. tuberculosis* H37Rv (Fig. 4b). Phylogenetic reconstruction highlighted distinct groups of *M. microti* strains that were linked either by their origin (human and wild-boar isolates) and/or by their spoligotype (‘llama’ vs ‘vole’). Indeed, *M. microti* strains showing a vole spoligotype that successfully infected humans (94-2272, Maus III and Maus IV) clustered separately from strains harbouring a llama spoligotype (ATCC 19422 and 35782, 416/01 and 8753/00) and from strains infecting wild boars (G25821 to G25828) (Fig. 4b).

### Virulence evaluation of *M. microti* strains in SCID mice

**Fig. 5.**
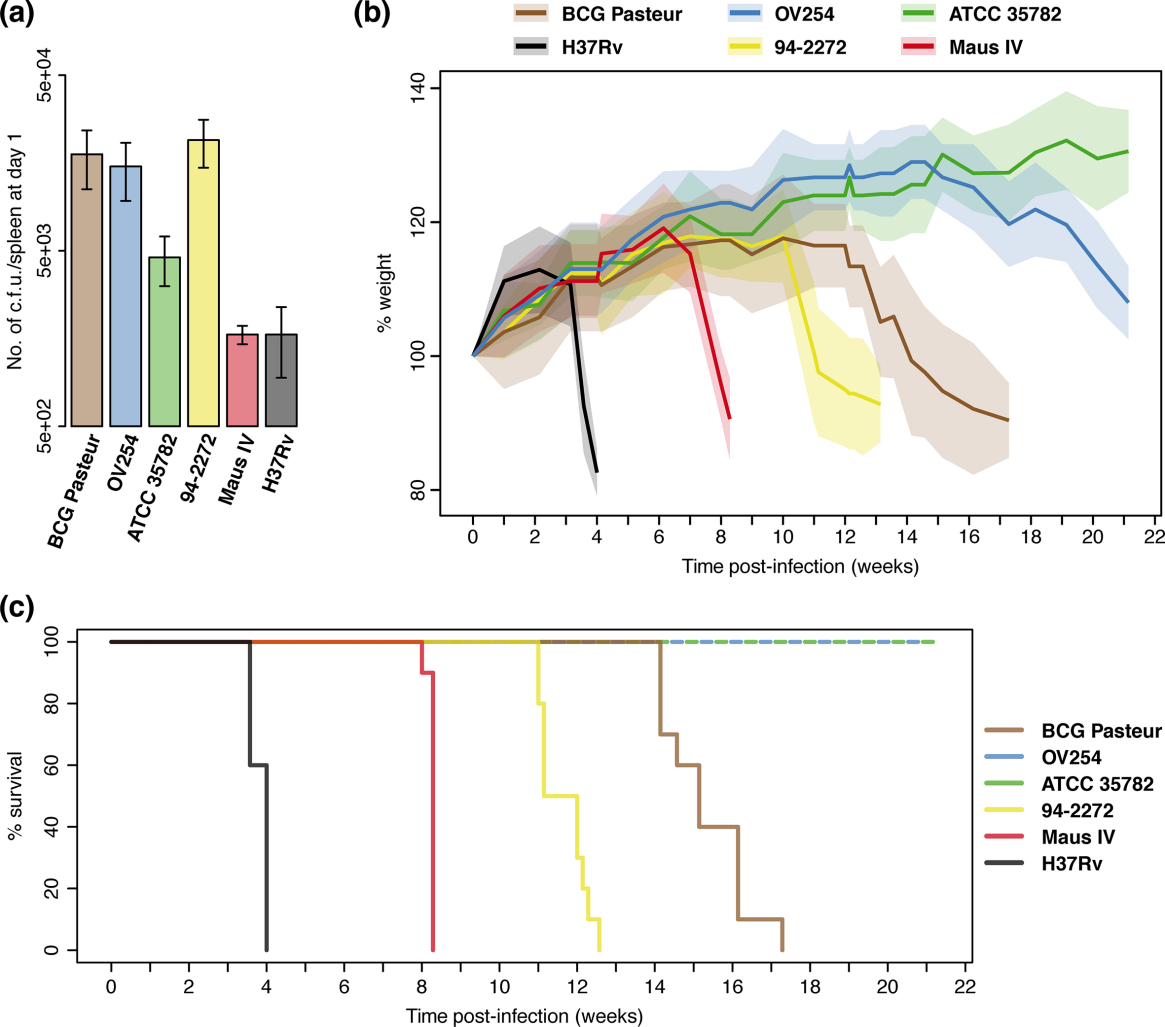
Virulence evaluation of *M. microti* strains relative to the control strains BCG Pasteur and *M. tuberculosis* H37Rv in SCID mice. A total of 13 SCID mice per group were infected intravenously via the lateral tail vein with 1×10^6^ c.f.u. in 200 µl of PBS (1x). (a) Bacterial load in the spleen of infected mice after 1 day of infection. *N*=3 mice per group. The results are shown as the mean±sd. (b) Percentage of weight change of infected SCID mice over time. *N*=10 mice per group. Coloured lines and surrounding areas represent the mean±sd. (c) Percentage of survival of infected SCID mice over time. *N*=10 mice per group. Mice were killed when reaching the humane endpoint, defined as a weight loss of more than 20 %, in accordance with ethics committee guidelines.

For exploring the virulence of four selected *M. microti* strains (OV254, ATCC 35782, 94-2272 and Maus IV) relative to *M. tuberculosis* H37Rv and BCG Pasteur control strains, we used an infection assay of SCID mice, as this model provides a rapid and sensitive method for evaluating the *in vivo* growth characteristics of mycobacterial strains during the acute phase of infection [31, 87–90]. This approach showed that all four tested *M. microti* strains were less virulent than *M. tuberculosis* H37Rv, as mice infected with the latter strain reached the humane endpoint (weight loss of more than 20 %) about 3–4 weeks post-infection (median time 28 days), when mice infected with *M. microti* or BCG strains were still well and gaining weight (Fig. 5). However, in the following weeks, a clear separation occurred between the two tested human clinical *M. microti* isolates Maus IV and 94-2272 on one hand, and the vole isolates OV254 and ATCC 35782 on the other hand. Indeed, mice infected with Maus IV reached the humane endpoint at about 8 weeks post-infection (median time 58 days; log-rank test vs H37Rv *P* <10^−4^), followed by strain 94-2272-infected mice at 11–12 weeks post-infection (median time 81 days; log-rank test vs H37Rv *P* <10^−4^), whereas mice infected with the BCG Pasteur control strain reached the humane endpoint starting from 14 weeks post-infection (median time 106 days; log-rank test vs Maus IV or 94-2272 *P* <10^−4^) (Fig. 5). By contrast, mice infected with strains OV254 or ATCC 35782 did not reach the humane endpoint during the experiment and were still in good health 21 weeks post-infection when the experiment was stopped (log-rank test vs BCG Pasteur *P* <10^−5^) (Fig. 5). These results were similar to observations obtained by a preliminary, pilot experiment using a slightly different set of strains and fewer mice (Fig. S6). We conclude from the combined data that *M. microti* strains, including clinical isolates, showed lower virulence than the *M. tuberculosis* H37Rv control in the highly susceptible SCID mouse model, whereby the strongest attenuation was observed for the OV254 and ATCC 35782 vole isolates (Fig. 5). This finding is of particular importance as it confirms the safety of these strains even in immunodeficient hosts and thereby encouraged us to conduct additional preclinical vaccination studies with one of these strains.

### Protective efficacy of the highly attenuated *M. microti* ATCC 35782 strain in a preclinical vaccination model

**Fig. 6.**
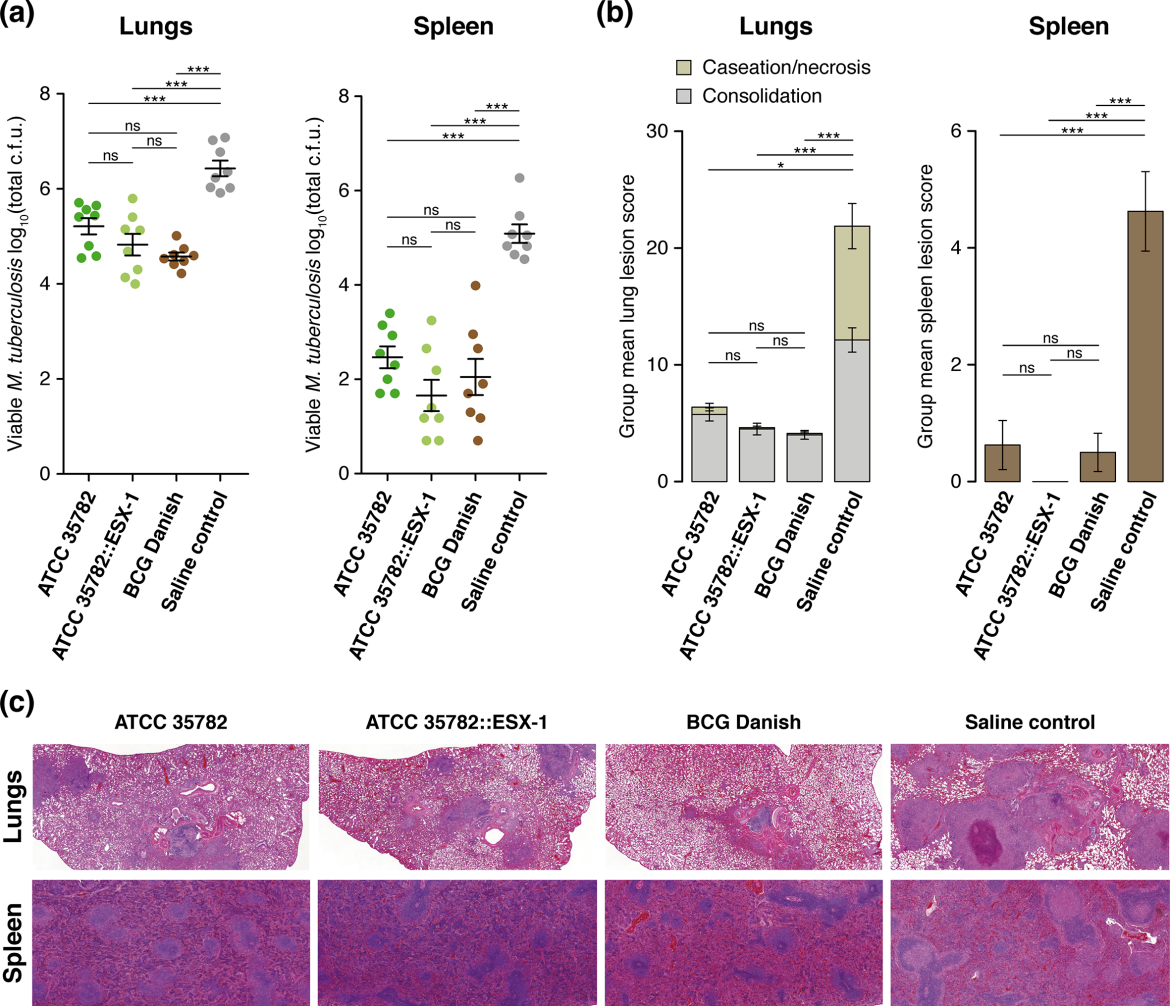
Protective efficacy of the vaccine candidates in the guinea pig low-dose infection model. Eight guinea pigs per group were vaccinated once subcutaneously on the nape with 5×10^4^ c.f.u. in 250 µl of PBS (1x) and challenged 12 weeks after immunization by the aerosol route with 10–20 c.f.u. *M. tuberculosis */lungs. Bacterial load (a), histological analysis of consolidation (b), and representative photomicrograph images of group mean lesion pathology from sections (c) of lungs and spleen of guinea pigs vaccinated with *M. microti* strains ATCC 35782, ATCC 35782::ESX-1 or BCG Danish or unvaccinated (saline control) after 4 weeks of infection with *M. tuberculosis *. (a) *N*=8 guinea pigs per group; mean±sem. One-way ANOVA test followed by Tukey HSD post-hoc test: ns, not significant; ***, *P* <0.001. (b) *N*=8 guinea pigs per group; mean±sem. Kruskal–Wallis test followed by Dunn post-hoc test: ns, not significant; *, *P* <0.05; ***, *P* <0.001. (c) Haematoxylin and eosin stained tissue sections of formalin-fixed and paraffin-embedded lung (×15 objective) and spleen (×25 objective).

From some older reports in the scientific literature, we know that the *M. microti* M.P. Prague strain (ATCC 35872; Fig. S1) was used successfully in the 1960s in former Czechoslovakia for large-scale human vaccination trials, where it showed a protective efficacy similar to that of BCG [37]. However, with the improvement of the TB situation in Europe and political changes since then, this vaccine strain has nowadays almost been forgotten. Given the strong attenuation and harmlessness of ATCC 35872 in SCID mice observed here, as well as the availability of the complete genome sequence that we have generated, it was of particular interest to evaluate the protective efficacy conferred by this strain in the established low-dose aerosol guinea pig model, a preclinical animal model that is often used for classifying the protective efficacy of TB vaccine candidates in head-to-head comparison studies in the framework of European TB vaccine consortia [79, 91]. In this experiment, which lasted for 16 weeks, we also included an ESX-1 complemented version of the strain ATCC 35782, named ATCC 35872::ESX-1, in addition to BCG Danish-vaccinated and unvaccinated (saline) control animals. As shown in Fig. 6(a), vaccination with strains ATCC 35782, ATCC 35782::ESX-1 and BCG Danish induced significant protection against *M. tuberculosis* as compared to animals that had received saline controls (one-way ANOVA test: lungs, *P*=9.2×10^−8^; spleen, *P*=1.6×10^−8^). Accordingly, the mean histopathological scores, based on the number and severity of lesions, observed in lungs and spleens of vaccinated animals was reduced as compared to unvaccinated animals (Fig. 6b, c) (Kruskal–Wallis test: lungs, *P*=6.7×10^−7^; spleen, *P*=3.6×10^−5^). These results emphasize the potential of strain ATCC 35782 to protect against an *M. tuberculosis* challenge in the sensitive guinea pig model. As indicated by the c.f.u. counts obtained from lungs and spleens, the protective efficacy of *M. microti* ATCC 35782 against TB was equivalent to that of BCG (Fig. 6a; Tukey HSD post-hoc test *P* >0.05). Given the much stronger attenuation of ATCC 35782 in comparison to BCG in the SCID mouse virulence model, the significant protection conferred by strain ATCC 35782 relative to unvaccinated controls is still of particular interest for vaccination strategies in immunocompromised hosts, where BCG is contra-indicated [92].

## Discussion

In this work, we have described and analysed the complete genome sequences of five *M. microti* strains, from both human and vole origin, and have tested the protein secretion, virulence and vaccine potential of selected strains. While *M. microti* has traditionally been associated with voles, due to the work of Dr Wells and subsequent reports [3, 5, 93], the number of mammalian hosts from which *M. microti* strains have been isolated has continuously grown in recent years, thereby allowing certain *M. microti* strain subtypes to be detected that are specifically associated with particular hosts.

Within the MTBC, *M. microti* is the most closely related to *Mycobacterium pinnipedii* strains, with one main difference, which is the specific absence of the RD1^mic^ genomic region from *M. microti* strains, and the presence of this region in *M. pinnipedii* [7, 30, 94]. Most interestingly, RD1^mic^ overlaps in part with the region of difference 1 (RD1^BCG^) absent from BCG strains [33, 34, 95]. In the absence of inter-strain recombination events, the deletion of a non-repetitive region of the chromosome cannot be repaired and, thus, will characterize all the descendants of the bacterial clone in which the deletion occurred [7, 94]. Thus, the RD1^mic^ deletion represents a reliable genotypic marker that links all strains showing this characteristic genomic mark to a recent common ancestor [7]. Hence, our first objective was to follow up the evolutionary events that happened since this bottleneck and determine by which degree *M. microti* isolates of human origin differed from the vole isolates, including those that were previously used as live attenuated vaccines against TB.

As outlined in Results and Fig. 1(a), the *in silico*-generated genome-wide IS*6110* insertion map indicated the variable presence of 8 to 15 IS*6110* elements in the tested strains, with several common positions shared between some strains. The three clinical *M. microti* isolates showed the greatest overlap of their IS*6110* insertion profiles, pointing to a close genomic relationship, which was also observed in the gene content and gene arrangements inside the polymorphic CRISPR-Cas and phospholipase C-PPE38 region (Figs 1b and 2a). Indeed, the clinical isolates all lacked the genes encoding Cas1, Cas2 and part of Csm6 protein in the CRISPR-Cas locus, pointing to likely ancestral homologous recombination events involving IS*6110* elements in a common progenitor strain. The presence of *cas1*, *cas2* and *csm6* genes in the ATCC 35782 vaccine strain suggests that this truncation of the CRISPR-Cas locus in clinical strains occurred in a subgroup of *M. microti* strains, and that the common progenitor strain in which the deletion of the RD1^mic^ region had occurred still carried a full CRISPR-Cas gene set, similar to *M. tuberculosis *. Moreover, the presence of certain spacers in the DR region, used for generating spoligotyping profiles, did not necessarily correlate with the host species, as ATCC 35782, which is described as a vole isolate (OV166) [37], carries more spacers (corresponding to the so-called llama spoligotype profile) than the human isolates and the OV254 strain, which all show a vole spoligotype profile with only the second spacer cluster present. This finding is consistent with the variety of different spoligotypes observed for *M. microti* strains isolated from wild boars [9] and underlines that spoligotype information does not always represent a reliable marker for phylogenetic reconstruction.

Another such polymorphic region in MTBC genomes is the *plcABC-ppe38* locus, which corresponds to the RD5 region (Fig. 2a). Like the above-mentioned CRISPR-Cas DR region, the phospholipase C-encoding genes *plcA, plcB*, *plcC* and *plcD* also represent a preferential target for IS*6110* transposition [96] and, hence, are associated with frequent insertion- and deletion-causing recombination events, as can be seen for strain OV254. While in previous work we have speculated that the presence or absence of certain of these four *plc* genes might have an impact on the level of virulence of *M. microti* [30], a hypothesis which was based on a previous report on *M. tuberculosis* Mt103 mutants [97], more recent studies did not confirm an impact of *plc* genes on the virulence of *M. tuberculosis* H37Rv and Mt103 [98]. Our observation that *M. microti* ATCC 35782 and the three tested clinical *M. microti* strains all harbour an intact *plcABC* gene cluster (Fig. 2a), but differ strongly in virulence, further dismisses the hypothesis of a putative impact of *plc* genes in the virulence of MTBC members. However, *plcABC* are not the only variable genes in this RD5^mic^ locus, as it also includes the *ppe38*, *ppe71*, *esxX* and *esxY* genes. Whereas all human isolates had this four-gene cluster intact, vole isolate ATCC 35782 has undergone a common recombination event between the almost identical *ppe38* and *ppe71* genes, known as an RvD7 deletion, that results in the effective deletion of *ppe38*, *esxX* and *esxY* [84]. Concurrent with previous work, we found that this single *ppe38*/*71* copy was sufficient for functional PE_PGRS secretion in ATCC 35782, but that the RD5^mic^ deletion in OV254 abrogated PE_PGRS secretion (Fig. 2b), similar to other RD5 deletions in the MTBC [74]. Complementation of OV254 with the four-gene *ppe38-71* locus of CDC1551 resulted in high levels of PE_PGRS secretion. Interestingly, the human isolate Maus III showed little to no functional PE_PGRS secretion despite having a full *ppe38-71* locus. This finding is reminiscent of the strongly decreased PE_PGRS secretion and PPE-MPTR immunogenicity reported for lineage L5.2 strains of *M. africanum* [72], pointing to the existence of additional polymorphisms influencing PE_PGRS and PPE-MPTR secretion in these strains to be identified by future research.

Overall, our findings suggest that the RD1^mic^-deleted common ancestor strain of the *M. microti* clade had the CRISPR-Cas and the *plcABC-ppe38* loci intact, before strain- or group-specific deletions occurred. These results are also in good agreement with the phylogenetic reconstruction, which show that the three tested human isolates from various geographical origin clustered together in the phylogenetic tree (Fig. 4b). Importantly, the human isolates also showed higher virulence than the vole isolates in the SCID mouse model of infection, while being still much lower than that of the *M. tuberculosis* H37Rv control (Fig. 5). The overall lower virulence of *M. microti* strains, relative to the *M. tuberculosis* control can be explained by the absence of the ESX-1 secretion system in *M. microti* strains due to the deletion of the RD1^mic^ region from all *M. microti* strains, in analogy to attenuated *M. tuberculosis* strains lacking the ESX-1 secretion systems [99]. The absence of ESX-1 functions was also judged as being one of the key attenuating factors of *M. bovis* BCG [31, 88]. Based on the results of the SCID mouse experiments, we can add that the absence of a functional ESX-1 system is also very likely one of the key factors of the attenuation of the previously used *M. microti* M.P. Prague vaccine strain (ATCC 35782). However, as suggested by the enhanced virulence of the three tested ESX-1-deficient clinical *M. microti* isolates, absence of ESX-1 might not represent the only factor contributing to the strong attenuation of the ATCC 35782 and OV254 vole isolates.

These data are consistent with observations from investigations on the BCG vaccine strains, which apart from the loss of the RD1 region also carry additional attenuating mutations, like frameshift mutations in the *pstB* and *phoT* genes, encoding key components of the mycobacterial high-affinity phosphate-uptake system [49, 100]. Such mutations likely have accumulated during the 13 years of passaging of the original *M. bovis* strain on potato-ox-bile medium that Calmette and Guérin initially used [101], or during the more than 1000 passages in different research institutions, which today characterize the various BCG sub-strains [49, 102]. Interestingly, for the *M. microti* M.P. Prague vaccine, similar events appear to have happened. As outlined in their report from 1976, Sula and Radovsky described the *M. microti* strain that they initially received from Dr Wells (OV166) as too virulent for their vaccination purposes and, thus, had subjected the strain to additional *in vitro* passaging, after which the strain was considered as safe for use as a human vaccine [37, 38]. It is likely that during these *in vitro* passages additional attenuating mutations were introduced into the strain, which also seem to contribute to the strong attenuation of ATCC 35782 in SCID mice, where it was more attenuated than BCG (Fig. 5).

As another perspective, the observed higher virulence of the human *M. microti* isolates relative to the vole *M. microti* strains confirms that under certain conditions ESX-1-deprived strains may still act as pathogens in susceptible hosts. These observations are consistent with the fact that several members of the MTBC are natural ESX-1 deletion mutants and still occupy an important role as natural animal pathogens [1]. Besides *M. microti *, this is the case for '*Mycobacterium mungi*' [103], the dassie bacillus [104] and '*Mycobacterium suricattae*' [105], which appear to be closely related to the human *M. africanum* L6 clade of the MTBC [46]. Whether this situation is linked to specific host factors that increase the susceptibility of certain hosts or individuals to infection with ESX-1-deprived MTBC strains still remains to be addressed. From studies on human patients who became victims of generalized BCG infections (BCGitis), some key host factors essential for efficient control of mycobacterial infections, such as IFN-γ and IL-12 receptors, have been identified [106]; however, it is not known if any of these factors play a role in the natural infection cycle of voles with *M. microti *, or mongoose or meerkats with '*M. mungi*' and '*M. suricattae*', respectively. Alternatively, the natural infection routes with ESX-1-deprived strains in wildlife may differ strongly from the aerosolic spread of *M. tuberculosis* in humans [107].

Finally, we also tested the ATCC 35782 vaccine strain in a standard guinea pig vaccination model and found that this strain induced a protection level in the vaccinated guinea pigs that was significantly better than that observed for unvaccinated control animals, while being equivalent to the one induced by BCG Danish (Fig. 6). Taken together, we conclude that this strain, which has been used previously for the vaccination of more than half a million infants in former Czechoslovakia in the 1960s, still retains a substantial vaccine potential that might be worth reconsideration under some specific situations. Given the very high level of attenuation observed for this live vaccine strain in the hypersusceptible SCID mouse model, the *M. microti* ATCC 35782 strain might be an ideal candidate for vaccination of immunodeficient individuals, such as HIV-positive children and adults for whom BCG vaccination is not recommended [108, 109], but who might benefit the most from an immunization against TB.

In conclusion, we show here that *M. microti* harbours a substantial amount of diversity within its clade in addition to the common deletion of the RD1^mic^ region. The combined findings of our study and those of previous analyses [46] suggest that the diversification since this RD1^mic^-associated evolutionary bottleneck has created several sub-clades of strains infecting different mammalian species, where they may maintain natural infection cycles. Given previous findings that *M. microti* may infect a wider range of mammalian hosts than originally thought [7, 21], additional genome analysis from a larger number of animal isolates will further fine-characterize the phylogenomic structure of the *M. microti* clade. From analysis of the current literature, it also seems clear that human cases caused by *M. microti* remain an exceptional phenomenon. Thus, it is of importance to emphasize that the three strains isolated from human patients included in our study cluster in a separate group of the phylogenetic tree, suggesting that both mycobacterial-strain-related factors and host-related factors might contribute to turn ESX-1-deleted *M. microti* strains into human pathogens. Whether in these special and rare cases humans might serve as a spillover host, similar to situations where other animal-adapted MTBC members, such as *M. pinnipedii* or *M. bovis *, may cause sporadic zoonotic human disease [1, 110–112], remains an open question, which is difficult to answer given the small number of available clinical isolates of *M. microti *. The full genome sequences of these strains provided here, thus, opens new perspectives for comparative analyses with larger datasets, as soon as they become available. Until then, we should consider *M. microti* strains as a clade of ESX-1-deficient MTBC strains with a substantial variability of virulence that ranges from completely attenuated members, such as the ATCC 35782 vaccine strain, to quite highly virulent strains, such as the Maus IV clinical isolate.

## Supplementary Data

Supplementary material 1

Supplementary material 2
