# Supplementary material for: Pathogenomic analyses of Mycobacterium microti, an ESX-1-deleted member of the Mycobacterium tuberculosis complex causing disease in various hosts
Source: Microb Genom. 2021 Feb 2;7(2):000505. doi: 10.1099/mgen.0.000505 (PMC8208694; doi:10.1099/mgen.0.000505)
Supplement: Supplementary material 1 [file mgen-7-505-s001.pdf]

ATCC™ | THE ESSENTIALS OF LIFE SCIENCE RESEARCH GLOBALLY DELIVERED™ take a tour

ABOUT | CULTURES AND PRODUCTS | SCIENCE | STANDARDS | DEPOSIT SERVICES

ATCC Advanced Catalog Search » **Product Details**

Product Description

Before submitting an order you will be asked to read and accept the terms and conditions of ATCC's Material Transfer Agreeme
Customers in Europe, Australia, Canada, China, Hong Kong, India, Israel, Japan, Korea, Macau, Mexico, New Zealand, Singap
information and to place an order for ATCC cultures and products.

Bacteria

**ATCC® Number:**	**35782™** Order this Item
**Organism:**	*Mycobacterium microti* Reed
**Designations:**	TMC 1608 [M.P. Prague]
**Isolation:**	field vole
**Depositor:**	Trudeau Mycobacterial Culture Collection - TMC
**History:**	ATCC <<--Trudeau Mycobacterial Culture Collection - TMC<<--L. Sula <<--- A.Q. Wells
Biosafety Level:	2
**Shipped:**	freeze-dried
**Growth Conditions:**	ATCC medium90: Lowenstein Jenson medium<br>**Temperature:** 37.0°C
**Permits/Forms:**	In addition to the MTA mentioned above, other ATCC and/or regulatory permits may be required for the transfer of this ATCC material. Anyone purchasing ATCC material is ultimately responsible for obtaining the permits. Please click here for information regarding the specific requirements for shipment to your location.

Figure S1. Description of the *M. microti* M.P. Prague strain retrieved from the American Type Collection previously available under the name ATCC 35782.

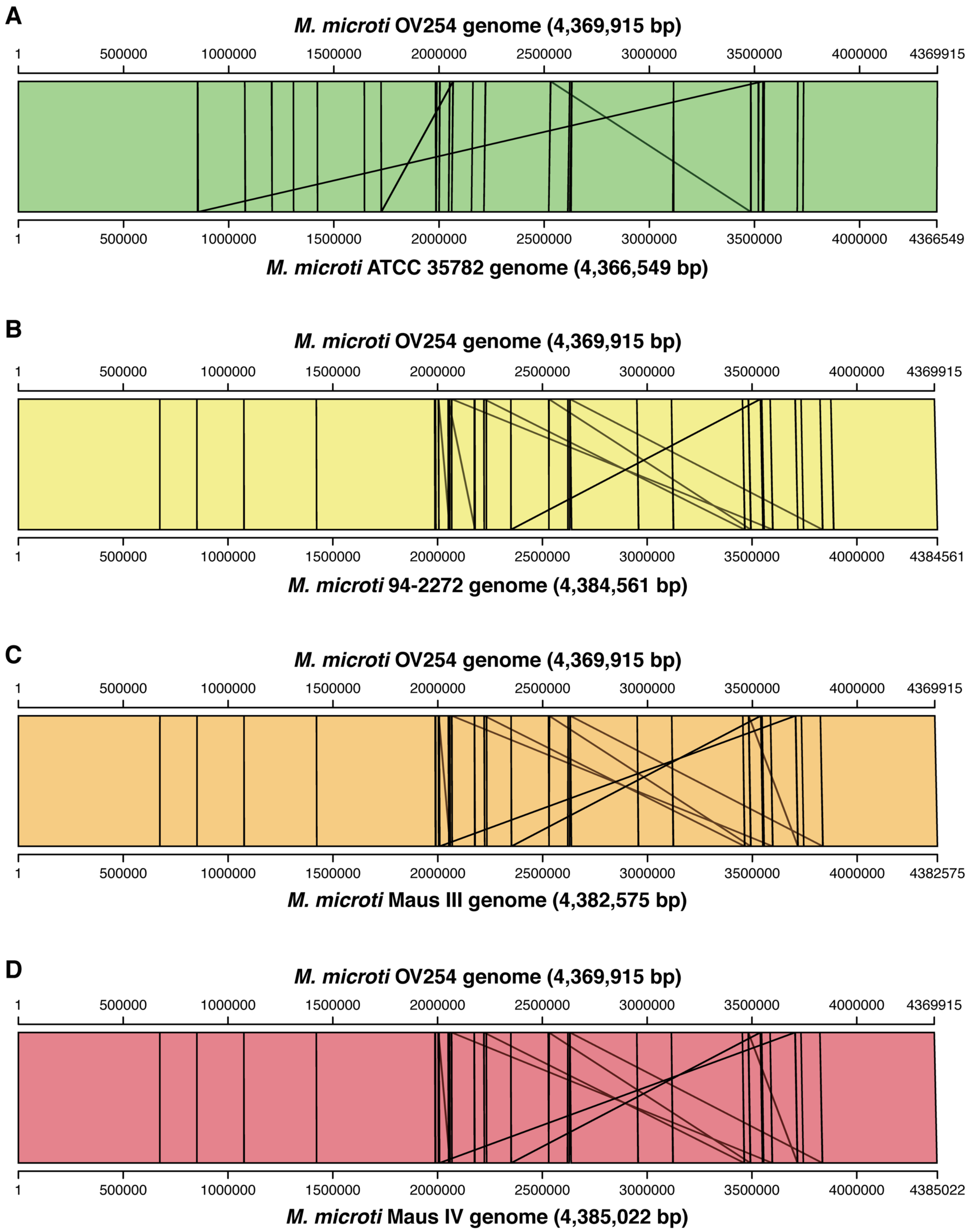

Figure S2. Pairwise comparison between *M. microti* strains OV254 and ATCC 35782 (**A**), 94-2272 (**B**), Maus III (**C**), or Maus IV (**D**) genome sequences. Only BLAST hits with at least 95% of identities are shown.

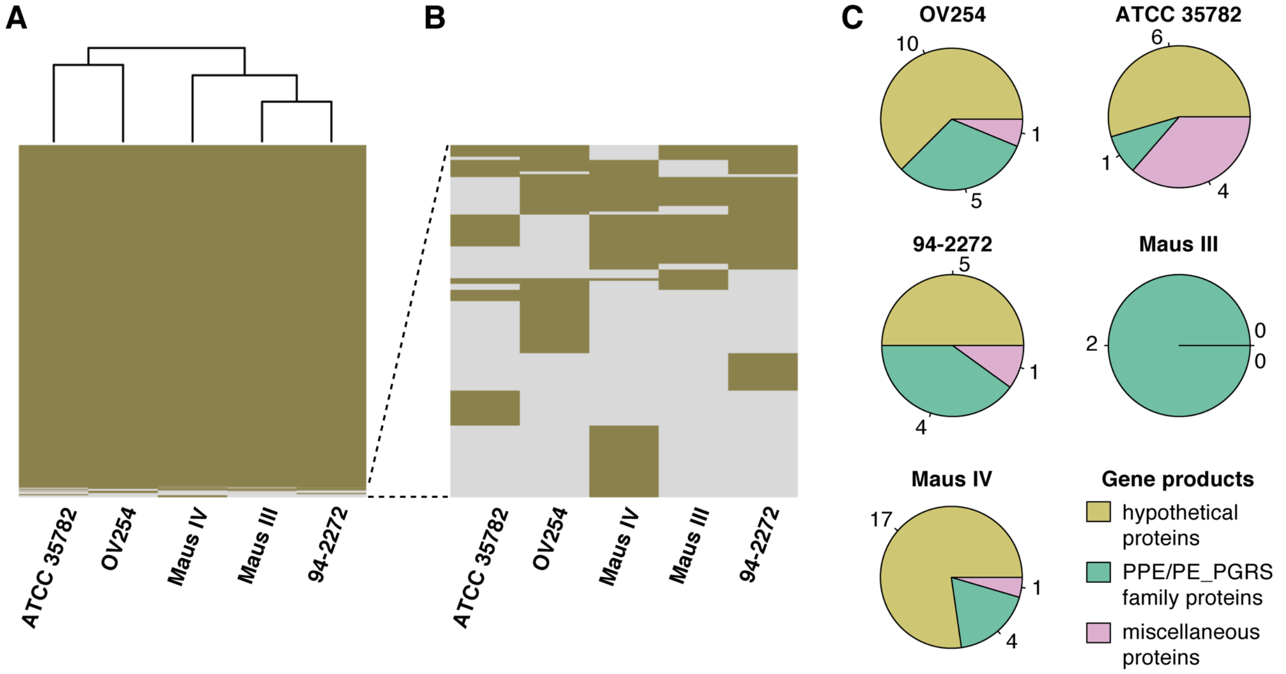

Figure S3. Gene synteny within *M. microti* strains. (**A**,**B**) Hierarchical clustering of orthologous genes in *M. microti* strains. Genes present and absent are depicted in brown and grey, respectively. (**B**) is a higher magnification of the bottom part of (**A**). (**C**) Annotated product of non-orthologous genes for each *M. microti* strain.

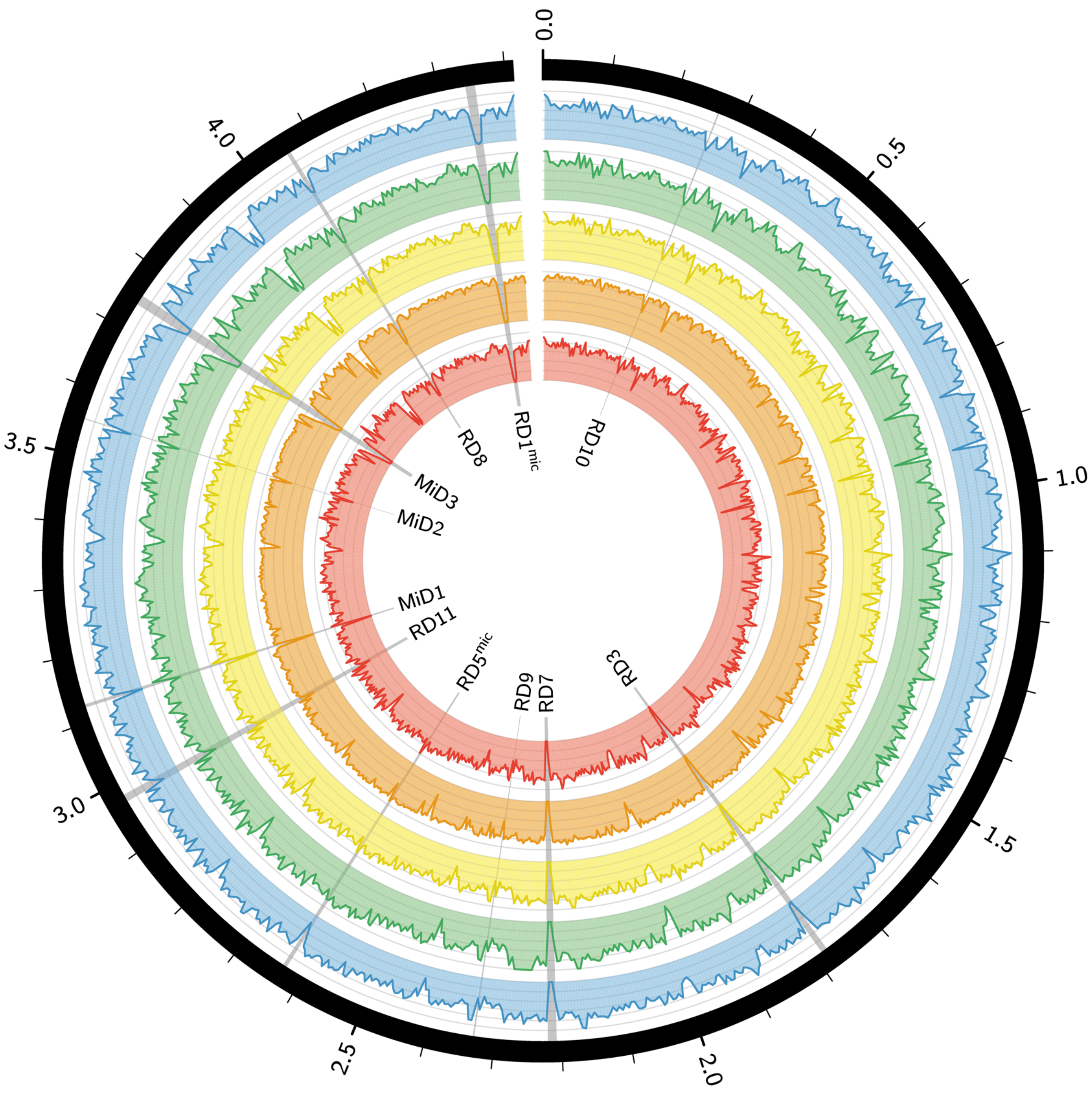

Figure S4. Coverage of sequencing reads from *M. microti* strains mapped against the genome of *M. tuberculosis* H37Rv. Read density was calculated in 5-kb non-overlapping windows for *M. microti* strains OV254 (blue), ATCC 35782 (green), 94-2272 (yellow), Maus III (orange) and Maus IV (red) following mapping against *M. tuberculosis* H37Rv genome (black). Regions of difference (RDs and MiDs) previously linked to *M. microti* clade are highlighted in grey.

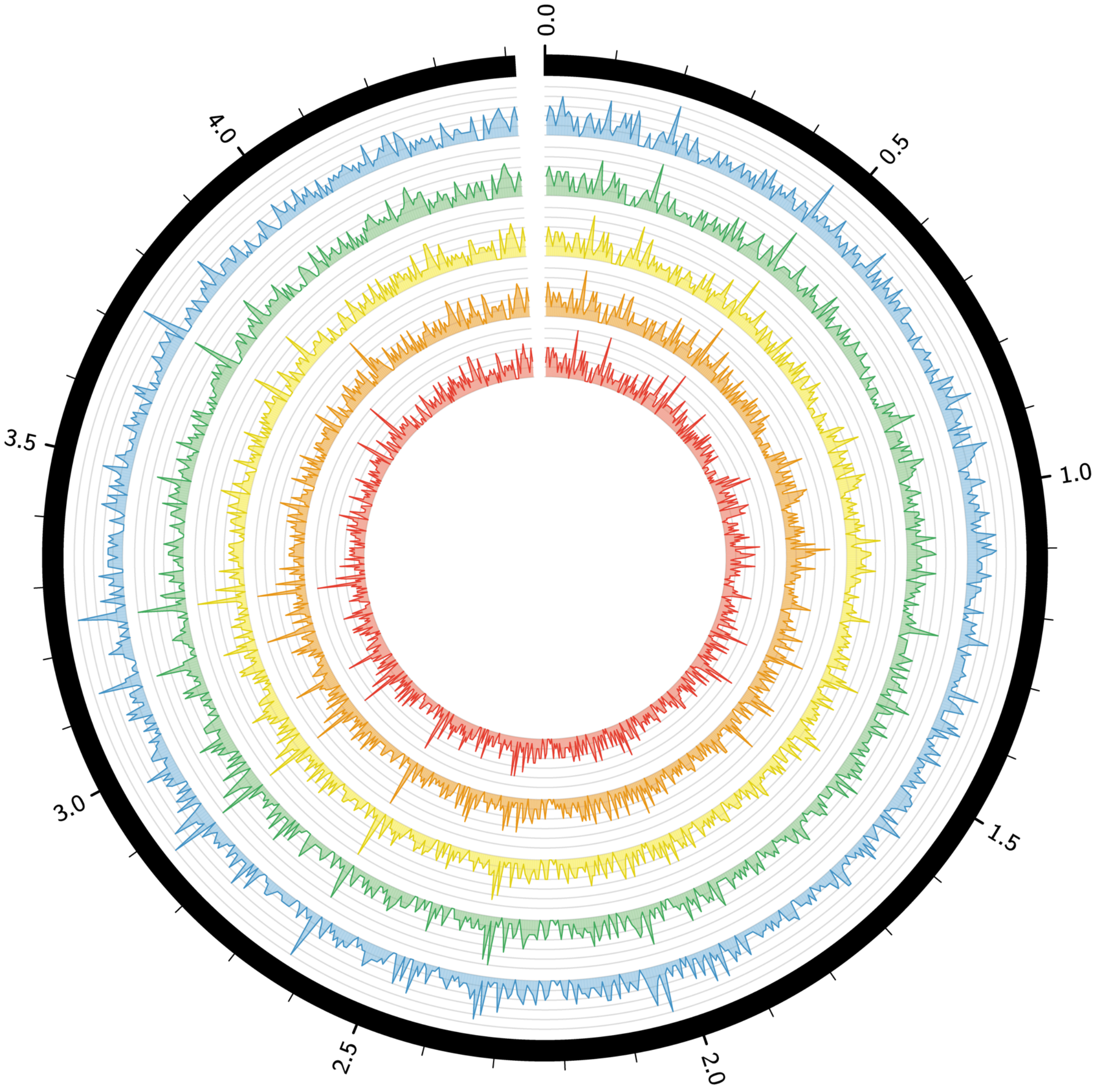

Figure S5. Called variants in *M. microti* strains relative to *M. tuberculosis* H37Rv. Density of detected variants was calculated in 5-kb non-overlapping windows for *M. microti* strains OV254 (blue), ATCC 35782 (green), 94-2272 (yellow), Maus III (orange) and Maus IV (red) following mapping of sequencing reads against *M. tuberculosis* H37Rv genome (black).

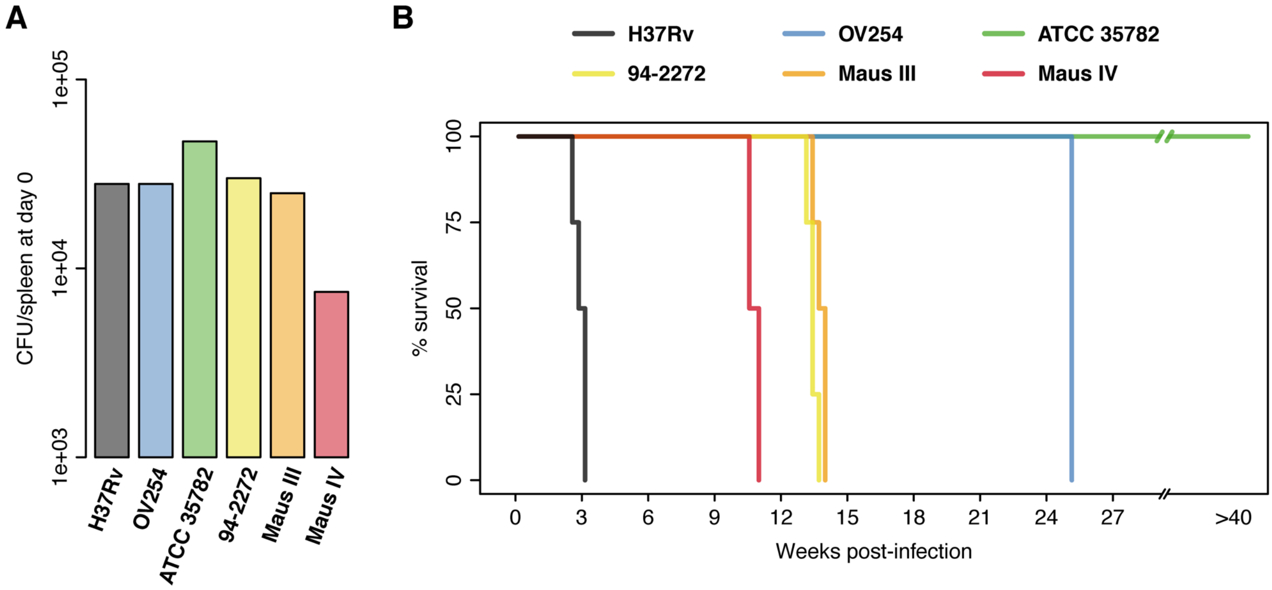

Figure S6. Pilot virulence assay of *M. microti* strains relative to the control strain *M. tuberculosis* H37Rv in SCID mice. 5 SCID mice per group were infected intravenously via the lateral tail vein with 1x10^6 CFU in 200 µl. (**A**) Bacterial load in spleen of infected SCID mice at the day 0 of infection. N = 1 mouse per group. (**B**) Percentage of survival of infected SCID mice over time. N = 4 mice per group. Mice were killed when reaching the humane endpoint, defined as a weight loss of more than 20% in accordance with ethics committee guidelines.
